# {μ-6,6′-Dimeth­oxy-2,2′-[ethane-1,2-diyl­bis(nitrilo­methyl­idyne)]diphenolato-1κ^4^ *O* ^6^,*O* ^1^,*O* ^1′^,*O* ^6′^:2κ^4^ *O* ^1^,*N*,*N*′,*O* ^1′^}(methanol-1κ*O*)(perchlorato-1κ*O*)nickel(II)sodium

**DOI:** 10.1107/S1600536809007776

**Published:** 2009-03-14

**Authors:** Hui-Quan Xiao

**Affiliations:** a Department of Chemistry, Shaoxing University, Shaoxing 312000, People’s Republic of China

## Abstract

The mol­ecule of the title compound, [NaNi(C_18_H_18_N_2_O_4_)(ClO_4_)(CH_3_OH)], is almost planar, the maximum deviation from the molecular plane being 5.3 (1) Å. The Ni^2+^ ion is N_2_O_2_ coordinated by the Schiff base ligand, leading to a slightly distorted square-planar environment. The Na atom is chelated by the four O atoms of the Schiff base ligand and is coordinated by the O atoms of a methanol ligand and a perchlorate anion. The perchlorate ion is disordered over two sites with occupancies 0.723 (12):0.277 (12).

## Related literature

For background to Schiff bases as ligands for metal ions and their roles in biochemical processes, see: Lindoy *et al.* (1976 ▶). For the steric, electronic and lipophilic properties of *N*,*N*-disalicylideneethyl­enediamine type Schiff bases ligands, see: Correia *et al.* (2005 ▶). For bond-length data, see: Allen *et al.* (1987 ▶).
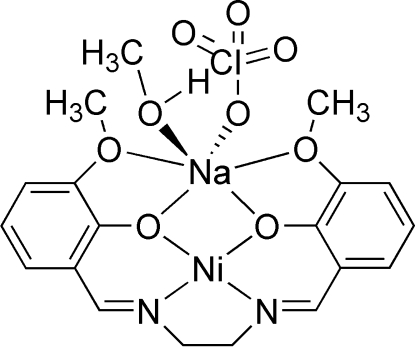


## Experimental

### 

#### Crystal data

[NaNi(C_18_H_18_N_2_O_4_)(ClO_4_)(CH_4_O)]
*M* *_r_* = 539.54
Monoclinic, 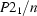

*a* = 12.026 (2) Å
*b* = 8.1360 (16) Å
*c* = 23.394 (5) Å
β = 104.302 (3)°
*V* = 2218.0 (8) Å^3^
*Z* = 4
Mo *K*α radiation
μ = 1.07 mm^−1^
*T* = 273 K
0.14 × 0.12 × 0.11 mm

#### Data collection

Bruker SMART CCD area-detector diffractometer
Absorption correction: multi-scan (*SADABS*; Bruker, 2000 ▶) *T*_min_ = 0.865, *T*_max_ = 0.892
10559 measured reflections
3906 independent reflections
3120 reflections with *I* > 2σ(*I*)
*R*_int_ = 0.023

#### Refinement

*R*[*F* ^2^ > 2σ(*F* ^2^)] = 0.053
*wR*(*F* ^2^) = 0.159
*S* = 1.06
3906 reflections
339 parameters
H-atom parameters constrained
Δρ_max_ = 1.03 e Å^−3^
Δρ_min_ = −1.14 e Å^−3^

### 

Data collection: *SMART* (Bruker, 2000 ▶); cell refinement: *SAINT* (Bruker, 2000 ▶); data reduction: *SAINT*; program(s) used to solve structure: *SHELXS97* (Sheldrick, 2008 ▶); program(s) used to refine structure: *SHELXL97* (Sheldrick, 2008 ▶); molecular graphics: *SHELXTL* (Sheldrick, 2008 ▶); software used to prepare material for publication: *SHELXTL*.

## Supplementary Material

Crystal structure: contains datablocks I, global. DOI: 10.1107/S1600536809007776/hg2479sup1.cif

Structure factors: contains datablocks I. DOI: 10.1107/S1600536809007776/hg2479Isup2.hkl

Additional supplementary materials:  crystallographic information; 3D view; checkCIF report

## Figures and Tables

**Fig. 1.**
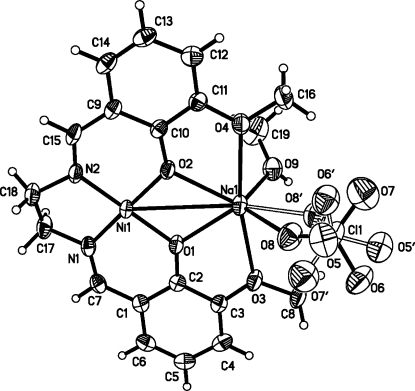
The independent molecules of the title compound, showing 30% probability displacement ellipsoids and the atom-numbering scheme.

## supplementary crystallographic information

### Comment

Schiff bases have been known as effective ligands for metal ions and used in the mechanism of many biochemical processes (Lindoy *et al.*, 1976). *N*,*N*-disalicylideneethylenediamine type Schiff bases ligands present versatile steric, electronic and lipophilic properties (Correia *et al.*, 2005). We report here the synthesis and crystal structure of the title compound (I). The molecular structure of (I) is shown in Fig.1. The values of the geometric parameters in (I) are normal (Allen *et al.*, 1987). Ni(II) and Na(I) are connected *via* two bridging oxygen atoms of the ligand. The six-coordinate Na atom adopts a distorted octahedral coordination geometry is completed by the O atoms derived from a perchlorate anion, while the four-coordinate Ni gives plane coordination.

### Experimental

A mixture of 6,6'-dimethoxy-2,2'-(ethane-1,2-diyldiiminodimethylene)diphenol (1 mmol) and nickel chloride (1 mmol) in absolute ethanol (15 ml) was stirred for 30 min and sodium perchlorate (1 mmol) was added, stirred for another 15 min and then filtered. The resulting clear orange solution was evaporated at room temperature for 7 days, after which large orange block-shaped crystals of the title complex suitable for X-ray diffraction analysis were obtained.

### Refinement

The H atoms were fixed geometrically and were treated as riding on their parent C atoms, with C—H distances in the range of 0.93–0.97 Å,or 0.82 Å (methanol hydroxyl) and with *U*_iso_(H) = 1.2*U*_eq_(parent atom), or *U*_iso_(H) = 1.5*U*_eq_(C_methyl_).

### Crystal data

**Table:**

[NaNi(C_18_H_18_N_2_O_4_)(ClO_4_)(CH_4_O)]	*F*(000) = 1111.9
*M**_r_* = 539.54	*D*_x_ = 1.616 Mg m^−^^3^
Monoclinic, *P*2_1_/*n*	Mo *K*α radiation, λ = 0.71073 Å
Hall symbol: -P 2yn	Cell parameters from 3601 reflections
*a* = 12.026 (2) Å	θ = 2.7–26.6°
*b* = 8.1360 (16) Å	µ = 1.07 mm^−^^1^
*c* = 23.394 (5) Å	*T* = 273 K
β = 104.302 (3)°	Block, orange
*V* = 2218.0 (8) Å^3^	0.14 × 0.12 × 0.11 mm
*Z* = 4	

### Data collection

**Table:**

Bruker SMART CCD area-detector diffractometer	3906 independent reflections
Radiation source: fine-focus sealed tube	3120 reflections with *I* > 2σ(*I*)
graphite	*R*_int_ = 0.023
φ and ω scans	θ_max_ = 25.0°, θ_min_ = 1.8°
Absorption correction: multi-scan (*SADABS*; Bruker, 2000)	*h* = −11→14
*T*_min_ = 0.865, *T*_max_ = 0.892	*k* = −9→9
10559 measured reflections	*l* = −27→27

### Refinement

**Table:**

Refinement on *F*^2^	Primary atom site location: structure-invariant direct methods
Least-squares matrix: full	Secondary atom site location: difference Fourier map
*R*[*F*^2^ > 2σ(*F*^2^)] = 0.053	Hydrogen site location: inferred from neighbouring sites
*wR*(*F*^2^) = 0.159	H-atom parameters constrained
*S* = 1.06	*w* = 1/[σ^2^(*F*_o_^2^) + (0.0852*P*)^2^ + 3.4175*P*] where *P* = (*F*_o_^2^ + 2*F*_c_^2^)/3
3906 reflections	(Δ/σ)_max_ = 0.001
339 parameters	Δρ_max_ = 1.03 e Å^−^^3^
0 restraints	Δρ_min_ = −1.14 e Å^−^^3^

### Special details

**Table:**

Geometry. All e.s.d.'s (except the e.s.d. in the dihedral angle between two l.s. planes) are estimated using the full covariance matrix. The cell e.s.d.'s are taken into account individually in the estimation of e.s.d.'s in distances, angles and torsion angles; correlations between e.s.d.'s in cell parameters are only used when they are defined by crystal symmetry. An approximate (isotropic) treatment of cell e.s.d.'s is used for estimating e.s.d.'s involving l.s. planes.
Refinement. Refinement of *F*^2^ against ALL reflections. The weighted *R*-factor *wR* and goodness of fit *S* are based on *F*^2^, conventional *R*-factors *R* are based on *F*, with *F* set to zero for negative *F*^2^. The threshold expression of *F*^2^ > σ(*F*^2^) is used only for calculating *R*-factors(gt) *etc*. and is not relevant to the choice of reflections for refinement. *R*-factors based on *F*^2^ are statistically about twice as large as those based on *F*, and *R*- factors based on ALL data will be even larger.

### Fractional atomic coordinates and isotropic or equivalent isotropic displacement parameters (Å^2^)

**Table:**

	*x*	*y*	*z*	*U*_iso_*/*U*_eq_	Occ. (<1)
Ni1	0.17531 (4)	0.91320 (6)	1.01069 (2)	0.0373 (2)	
Na1	0.14961 (15)	0.9105 (2)	0.86470 (7)	0.0501 (5)	
Cl1	0.00049 (13)	1.1323 (2)	0.73545 (7)	0.0721 (4)	
O1	0.2467 (2)	1.0034 (4)	0.95724 (11)	0.0448 (7)	
O2	0.0756 (2)	0.8289 (4)	0.94440 (12)	0.0447 (7)	
O3	0.3169 (3)	1.0958 (4)	0.86647 (14)	0.0576 (9)	
O4	−0.0318 (3)	0.7296 (4)	0.84088 (13)	0.0557 (8)	
O5	−0.0995 (6)	1.2219 (13)	0.7369 (3)	0.102 (3)	0.723 (12)
O6	0.0663 (7)	1.2518 (12)	0.7120 (4)	0.104 (3)	0.723 (12)
O7	−0.0350 (8)	1.0008 (11)	0.6966 (4)	0.128 (4)	0.723 (12)
O8	0.0599 (6)	1.0976 (10)	0.7922 (4)	0.090 (3)	0.723 (12)
O5'	0.0360 (19)	1.150 (3)	0.6850 (10)	0.102 (7)	0.277 (12)
O6'	−0.1060 (17)	1.082 (3)	0.7329 (9)	0.098 (7)	0.277 (12)
O7'	0.0321 (18)	1.264 (3)	0.7734 (10)	0.118 (9)	0.277 (12)
O8'	0.0764 (13)	1.002 (2)	0.7645 (8)	0.076 (5)	0.277 (12)
O9	0.2372 (3)	0.6892 (5)	0.83487 (16)	0.0696 (10)	
H9A	0.2928	0.6982	0.8209	0.12 (3)*	
N1	0.2807 (3)	0.9918 (5)	1.07592 (14)	0.0457 (9)	
N2	0.0964 (3)	0.8305 (5)	1.06239 (15)	0.0458 (9)	
C1	0.3996 (4)	1.1441 (5)	1.02507 (19)	0.0441 (10)	
C2	0.3391 (3)	1.0965 (5)	0.96838 (19)	0.0398 (9)	
C3	0.3795 (4)	1.1522 (5)	0.91995 (19)	0.0435 (10)	
C4	0.4748 (4)	1.2522 (6)	0.9279 (2)	0.0552 (12)	
H4	0.5003	1.2882	0.8956	0.066*	
C5	0.5326 (4)	1.2990 (6)	0.9852 (2)	0.0590 (12)	
H5	0.5965	1.3672	0.9908	0.071*	
C6	0.4969 (4)	1.2463 (6)	1.0322 (2)	0.0560 (12)	
H6	0.5368	1.2776	1.0699	0.067*	
C7	0.3659 (4)	1.0869 (6)	1.0762 (2)	0.0486 (11)	
H7	0.4096	1.1216	1.1127	0.058*	
C8	0.3589 (5)	1.1308 (8)	0.8156 (2)	0.0695 (15)	
H8A	0.3547	1.2470	0.8082	0.104*	
H8B	0.3130	1.0738	0.7821	0.104*	
H8C	0.4372	1.0952	0.8225	0.104*	
C9	−0.0578 (4)	0.6982 (5)	0.99182 (19)	0.0443 (10)	
C10	−0.0165 (3)	0.7390 (5)	0.94207 (18)	0.0409 (9)	
C11	−0.0784 (4)	0.6823 (6)	0.8863 (2)	0.0455 (10)	
C12	−0.1784 (4)	0.5926 (6)	0.8798 (2)	0.0562 (12)	
H12	−0.2192	0.5578	0.8427	0.067*	
C13	−0.2176 (4)	0.5547 (6)	0.9300 (3)	0.0593 (13)	
H13	−0.2844	0.4934	0.9260	0.071*	
C14	−0.1599 (4)	0.6058 (6)	0.9838 (2)	0.0555 (13)	
H14	−0.1878	0.5798	1.0164	0.067*	
C15	0.0024 (4)	0.7488 (6)	1.0494 (2)	0.0506 (11)	
H15	−0.0294	0.7200	1.0804	0.061*	
C16	−0.0870 (5)	0.6700 (9)	0.7830 (2)	0.0777 (17)	
H16A	−0.0888	0.5520	0.7834	0.117*	
H16B	−0.0451	0.7064	0.7553	0.117*	
H16C	−0.1640	0.7117	0.7715	0.117*	
C17	0.2655 (5)	0.9255 (7)	1.1318 (2)	0.0665 (15)	
H17A	0.2888	1.0066	1.1629	0.080*	
H17B	0.3126	0.8282	1.1428	0.080*	
C18	0.1423 (5)	0.8834 (7)	1.1241 (2)	0.0668 (15)	
H18A	0.1345	0.7955	1.1509	0.080*	
H18B	0.1003	0.9784	1.1326	0.080*	
C19	0.2246 (7)	0.5342 (9)	0.8564 (4)	0.106 (2)	
H19A	0.2947	0.5023	0.8838	0.159*	
H19B	0.2069	0.4572	0.8244	0.159*	
H19C	0.1634	0.5353	0.8761	0.159*	

### Atomic displacement parameters (Å^2^)

**Table:**

	*U*^11^	*U*^22^	*U*^33^	*U*^12^	*U*^13^	*U*^23^
Ni1	0.0411 (3)	0.0449 (3)	0.0281 (3)	0.0071 (2)	0.0126 (2)	0.0040 (2)
Na1	0.0508 (10)	0.0657 (12)	0.0362 (9)	0.0019 (8)	0.0151 (7)	0.0012 (8)
Cl1	0.0655 (9)	0.0880 (11)	0.0692 (9)	0.0145 (8)	0.0287 (7)	0.0277 (8)
O1	0.0419 (16)	0.0623 (19)	0.0320 (14)	−0.0024 (14)	0.0123 (12)	0.0025 (14)
O2	0.0444 (16)	0.0592 (19)	0.0349 (15)	−0.0049 (14)	0.0179 (12)	0.0009 (13)
O3	0.0554 (19)	0.080 (2)	0.0412 (17)	−0.0055 (17)	0.0197 (15)	0.0096 (16)
O4	0.0533 (18)	0.071 (2)	0.0439 (17)	−0.0065 (16)	0.0147 (14)	−0.0040 (16)
O5	0.088 (5)	0.121 (7)	0.094 (5)	0.030 (5)	0.017 (4)	−0.005 (5)
O6	0.101 (5)	0.116 (7)	0.101 (6)	−0.012 (5)	0.036 (5)	0.032 (5)
O7	0.136 (7)	0.116 (7)	0.125 (7)	0.020 (6)	0.016 (6)	−0.007 (6)
O8	0.081 (4)	0.105 (7)	0.077 (5)	0.005 (4)	0.008 (3)	0.025 (4)
O5'	0.097 (15)	0.12 (2)	0.091 (15)	0.004 (14)	0.027 (11)	0.013 (13)
O6'	0.087 (13)	0.115 (18)	0.090 (13)	0.003 (12)	0.020 (10)	0.012 (12)
O7'	0.112 (15)	0.123 (18)	0.112 (17)	0.006 (13)	0.015 (12)	0.003 (14)
O8'	0.072 (9)	0.088 (12)	0.067 (10)	0.006 (9)	0.015 (8)	0.010 (9)
O9	0.067 (2)	0.082 (3)	0.064 (2)	0.010 (2)	0.0246 (19)	−0.003 (2)
N1	0.051 (2)	0.055 (2)	0.0295 (17)	0.0113 (18)	0.0066 (15)	0.0046 (16)
N2	0.061 (2)	0.048 (2)	0.0331 (18)	0.0052 (18)	0.0199 (16)	0.0049 (16)
C1	0.044 (2)	0.044 (2)	0.043 (2)	0.0100 (19)	0.0077 (18)	0.0011 (19)
C2	0.036 (2)	0.040 (2)	0.043 (2)	0.0075 (17)	0.0101 (17)	0.0023 (18)
C3	0.042 (2)	0.045 (2)	0.044 (2)	0.0079 (19)	0.0119 (18)	0.0065 (19)
C4	0.052 (3)	0.047 (3)	0.072 (3)	0.004 (2)	0.024 (2)	0.013 (2)
C5	0.053 (3)	0.048 (3)	0.075 (3)	−0.006 (2)	0.013 (2)	0.000 (2)
C6	0.055 (3)	0.049 (3)	0.059 (3)	−0.003 (2)	0.005 (2)	−0.008 (2)
C7	0.050 (3)	0.054 (3)	0.037 (2)	0.007 (2)	0.0025 (19)	−0.0044 (19)
C8	0.075 (4)	0.093 (4)	0.049 (3)	−0.007 (3)	0.032 (3)	0.012 (3)
C9	0.046 (2)	0.045 (2)	0.048 (2)	0.0090 (19)	0.0224 (19)	0.0110 (19)
C10	0.039 (2)	0.042 (2)	0.046 (2)	0.0068 (18)	0.0190 (18)	0.0045 (19)
C11	0.039 (2)	0.051 (3)	0.049 (2)	0.0013 (19)	0.0151 (19)	0.002 (2)
C12	0.048 (3)	0.057 (3)	0.063 (3)	0.001 (2)	0.013 (2)	0.000 (2)
C13	0.045 (3)	0.061 (3)	0.078 (4)	−0.006 (2)	0.027 (3)	0.006 (3)
C14	0.051 (3)	0.056 (3)	0.069 (3)	0.007 (2)	0.034 (3)	0.014 (2)
C15	0.061 (3)	0.054 (3)	0.047 (3)	0.010 (2)	0.034 (2)	0.012 (2)
C16	0.080 (4)	0.106 (5)	0.046 (3)	−0.023 (3)	0.013 (3)	−0.016 (3)
C17	0.077 (4)	0.090 (4)	0.031 (2)	0.002 (3)	0.010 (2)	0.010 (2)
C18	0.092 (4)	0.076 (4)	0.038 (3)	−0.001 (3)	0.028 (3)	0.003 (2)
C19	0.114 (6)	0.092 (5)	0.117 (6)	0.042 (5)	0.038 (5)	0.034 (5)

### Geometric parameters (Å, °)

**Table:**

Ni1—O1	1.836 (3)	C1—C7	1.432 (6)
Ni1—N2	1.840 (4)	C2—C3	1.414 (6)
Ni1—N1	1.841 (4)	C3—C4	1.380 (6)
Ni1—O2	1.843 (3)	C4—C5	1.402 (7)
Ni1—Na1	3.3525 (19)	C4—H4	0.9300
Na1—O9	2.281 (4)	C5—C6	1.346 (7)
Na1—O1	2.318 (3)	C5—H5	0.9300
Na1—O8	2.335 (7)	C6—H6	0.9300
Na1—O2	2.351 (3)	C7—H7	0.9300
Na1—O8'	2.408 (16)	C8—H8A	0.9600
Na1—O3	2.506 (4)	C8—H8B	0.9600
Na1—O4	2.576 (4)	C8—H8C	0.9600
Cl1—O6'	1.33 (2)	C9—C14	1.412 (6)
Cl1—O5'	1.36 (2)	C9—C10	1.413 (6)
Cl1—O8	1.373 (7)	C9—C15	1.423 (7)
Cl1—O7'	1.39 (2)	C10—C11	1.410 (6)
Cl1—O7	1.400 (9)	C11—C12	1.383 (6)
Cl1—O5	1.414 (7)	C12—C13	1.403 (7)
Cl1—O6	1.445 (8)	C12—H12	0.9300
Cl1—O8'	1.456 (17)	C13—C14	1.343 (7)
O1—C2	1.315 (5)	C13—H13	0.9300
O2—C10	1.317 (5)	C14—H14	0.9300
O3—C3	1.370 (5)	C15—H15	0.9300
O3—C8	1.432 (5)	C16—H16A	0.9600
O4—C11	1.374 (5)	C16—H16B	0.9600
O4—C16	1.437 (6)	C16—H16C	0.9600
O9—C19	1.380 (8)	C17—C18	1.488 (8)
O9—H9A	0.8174	C17—H17A	0.9700
N1—C7	1.283 (6)	C17—H17B	0.9700
N1—C17	1.468 (6)	C18—H18A	0.9700
N2—C15	1.281 (6)	C18—H18B	0.9700
N2—C18	1.477 (6)	C19—H19A	0.9600
C1—C2	1.401 (6)	C19—H19B	0.9600
C1—C6	1.411 (7)	C19—H19C	0.9600
			
O1—Ni1—N2	176.62 (15)	C19—O9—H9A	113.8
O1—Ni1—N1	94.78 (15)	Na1—O9—H9A	122.5
N2—Ni1—N1	86.77 (17)	C7—N1—C17	119.5 (4)
O1—Ni1—O2	83.82 (12)	C7—N1—Ni1	126.8 (3)
N2—Ni1—O2	94.76 (15)	C17—N1—Ni1	113.5 (3)
N1—Ni1—O2	177.16 (14)	C15—N2—C18	118.5 (4)
O1—Ni1—Na1	41.38 (9)	C15—N2—Ni1	127.0 (3)
N2—Ni1—Na1	137.28 (13)	C18—N2—Ni1	114.0 (3)
N1—Ni1—Na1	135.89 (12)	C2—C1—C6	119.8 (4)
O2—Ni1—Na1	42.52 (9)	C2—C1—C7	120.8 (4)
O9—Na1—O1	112.26 (14)	C6—C1—C7	119.3 (4)
O9—Na1—O8	117.3 (3)	O1—C2—C1	124.2 (4)
O1—Na1—O8	120.1 (3)	O1—C2—C3	117.8 (4)
O9—Na1—O2	108.27 (15)	C1—C2—C3	118.0 (4)
O1—Na1—O2	63.49 (11)	O3—C3—C4	124.9 (4)
O8—Na1—O2	124.2 (2)	O3—C3—C2	113.8 (4)
O9—Na1—O8'	91.8 (5)	C4—C3—C2	121.3 (4)
O1—Na1—O8'	141.9 (5)	C3—C4—C5	119.3 (5)
O8—Na1—O8'	25.9 (4)	C3—C4—H4	120.4
O2—Na1—O8'	137.7 (4)	C5—C4—H4	120.4
O9—Na1—O3	92.89 (15)	C6—C5—C4	120.8 (5)
O1—Na1—O3	64.60 (11)	C6—C5—H5	119.6
O8—Na1—O3	81.1 (2)	C4—C5—H5	119.6
O2—Na1—O3	128.05 (13)	C5—C6—C1	120.9 (5)
O8'—Na1—O3	86.0 (4)	C5—C6—H6	119.5
O9—Na1—O4	85.03 (14)	C1—C6—H6	119.5
O1—Na1—O4	126.36 (12)	N1—C7—C1	125.6 (4)
O8—Na1—O4	90.0 (2)	N1—C7—H7	117.2
O2—Na1—O4	62.87 (11)	C1—C7—H7	117.2
O8'—Na1—O4	83.0 (4)	O3—C8—H8A	109.5
O3—Na1—O4	168.75 (13)	O3—C8—H8B	109.5
O9—Na1—Ni1	112.55 (11)	H8A—C8—H8B	109.5
O1—Na1—Ni1	31.57 (7)	O3—C8—H8C	109.5
O8—Na1—Ni1	130.2 (3)	H8A—C8—H8C	109.5
O2—Na1—Ni1	31.98 (8)	H8B—C8—H8C	109.5
O8'—Na1—Ni1	155.4 (5)	C14—C9—C10	119.1 (4)
O3—Na1—Ni1	96.18 (9)	C14—C9—C15	120.1 (4)
O4—Na1—Ni1	94.83 (8)	C10—C9—C15	120.8 (4)
O6'—Cl1—O5'	120.0 (14)	O2—C10—C11	117.8 (4)
O6'—Cl1—O8	104.1 (9)	O2—C10—C9	124.0 (4)
O5'—Cl1—O8	131.5 (10)	C11—C10—C9	118.2 (4)
O6'—Cl1—O7'	112.1 (14)	O4—C11—C12	124.9 (4)
O5'—Cl1—O7'	112.2 (16)	O4—C11—C10	113.6 (4)
O8—Cl1—O7'	62.9 (9)	C12—C11—C10	121.4 (4)
O6'—Cl1—O7	66.4 (11)	C11—C12—C13	119.1 (5)
O5'—Cl1—O7	68.2 (13)	C11—C12—H12	120.4
O8—Cl1—O7	118.2 (5)	C13—C12—H12	120.4
O7'—Cl1—O7	178.2 (9)	C14—C13—C12	120.8 (5)
O6'—Cl1—O5	49.2 (9)	C14—C13—H13	119.6
O5'—Cl1—O5	114.6 (10)	C12—C13—H13	119.6
O8—Cl1—O5	109.0 (5)	C13—C14—C9	121.4 (4)
O7'—Cl1—O5	71.7 (9)	C13—C14—H14	119.3
O7—Cl1—O5	106.6 (5)	C9—C14—H14	119.3
O6'—Cl1—O6	143.3 (10)	N2—C15—C9	125.9 (4)
O5'—Cl1—O6	43.8 (10)	N2—C15—H15	117.0
O8—Cl1—O6	107.6 (5)	C9—C15—H15	117.0
O7'—Cl1—O6	68.4 (10)	O4—C16—H16A	109.5
O7—Cl1—O6	112.1 (6)	O4—C16—H16B	109.5
O5—Cl1—O6	102.1 (6)	H16A—C16—H16B	109.5
O6'—Cl1—O8'	106.3 (12)	O4—C16—H16C	109.5
O5'—Cl1—O8'	100.9 (12)	H16A—C16—H16C	109.5
O8—Cl1—O8'	44.1 (7)	H16B—C16—H16C	109.5
O7'—Cl1—O8'	103.1 (12)	N1—C17—C18	108.5 (4)
O7—Cl1—O8'	78.5 (9)	N1—C17—H17A	110.0
O5—Cl1—O8'	143.6 (7)	C18—C17—H17A	110.0
O6—Cl1—O8'	109.3 (7)	N1—C17—H17B	110.0
C2—O1—Ni1	127.5 (3)	C18—C17—H17B	110.0
C2—O1—Na1	125.4 (3)	H17A—C17—H17B	108.4
Ni1—O1—Na1	107.04 (14)	N2—C18—C17	107.8 (4)
C10—O2—Ni1	127.4 (3)	N2—C18—H18A	110.1
C10—O2—Na1	127.1 (3)	C17—C18—H18A	110.1
Ni1—O2—Na1	105.50 (13)	N2—C18—H18B	110.1
C3—O3—C8	117.7 (4)	C17—C18—H18B	110.1
C3—O3—Na1	118.3 (3)	H18A—C18—H18B	108.5
C8—O3—Na1	123.6 (3)	O9—C19—H19A	109.5
C11—O4—C16	116.9 (4)	O9—C19—H19B	109.5
C11—O4—Na1	118.6 (3)	H19A—C19—H19B	109.5
C16—O4—Na1	124.5 (3)	O9—C19—H19C	109.5
Cl1—O8—Na1	150.8 (6)	H19A—C19—H19C	109.5
Cl1—O8'—Na1	135.7 (13)	H19B—C19—H19C	109.5
C19—O9—Na1	120.6 (4)		
